# Acute fibrinous and organizing pneumonia masquerading as a lower respiratory tract infection: a case report and review of the literature

**DOI:** 10.1186/s13104-015-0984-4

**Published:** 2015-02-10

**Authors:** Aftab Akhtar, Zain Ul Abideen

**Affiliations:** Department of Pulmonology and Critical Care, Shifa International Hospital, Islamabad, Pakistan; Resident Internal Medicine, Department of Internal Medicine, Shifa International Hospital, Islamabad, Pakistan

**Keywords:** Acute Fibrinous and Organizing Pneumonia (AFOP), Computerized Tomography (CT) guided biopsy

## Abstract

**Background:**

Acute Fibrinous and Organizing Pneumonia is a rare entity characterized by the histological pattern suggestive of diffuse alveolar damage, eosinophilic pneumonia and organizing pneumonia; the presence of intra alveolar “fibrin balls” distinguishes it from these conditions. Herein, we describe the association of acute fibrinous and organizing pneumonia with a respiratory tract infection. We believe that such an association has been extremely rarely described.

**Case presentation:**

We report the case of a 68 year old female patient of Afghan descent who presented with shortness of breath, cough and high grade fever not responding satisfactorily to standard antibiotic therapy. Imaging revealed bilateral basilar infiltrates and ground glass opacification of the right lower lung zone. Though the inflammatory markers decreased with antibiotic therapy, there was minimal improvement in the patient’s symptoms and radiological appearance of the lungs. Bronchoscopy was refused by the patient’s family and a Computed Tomography guided biopsy of the lung revealed a histological diagnosis of acute fibrinous and organizing pneumonia. Patient was initiated on high dose intravenous corticosteroid therapy followed by a maintenance dose of prednisolone at 40 mg/day. She recovered dramatically. However, due to poor compliance with treatment, she relapsed and was re-treated with the same regimen. Currently she is completely symptom free and is on a tapering corticosteroid dose.

**Conclusion:**

We conclude that AFOP may be a rare but under diagnosed entity and recommend that it should be considered in the differentials of a suspected pulmonary infection unresponsive to optimum antibiotic therapy.

## Background

Acute Fibrinous and Organizing Pneumonia (AFOP) was first described by Beasely et al. in 2002 as a distinct pattern of lung injury with histological analogy to diffuse alveolar damage, organizing pneumonia and eosinophilic pneumonia. The features that distinguish it histologically include intra alveolar fibrin balls, absence of typical hyaline membranes and eosinophils, and numerous foci of fibroblastic activity [1]. It has been described in all age groups with numerous associations including connective tissue and autoimmune diseases, drugs, occupational and environmental exposures, and less commonly infectious agents. Occasionally, no cause has been found [2].

The signs and symptoms are variable; two forms of the illness have been described; a severe form which leads to a rapid respiratory failure and a sub acute form, which has a good prognosis with treatment [1]. Diagnosis is by histological analysis of a biopsy obtained from the affected pulmonary tissue. There are no standard guidelines on treatment; steroids and immunosuppressants have been used with varying success rates, with best results in the sub acute form of the disease. Mechanical ventilation is often necessary for severe disease and carries poor prognosis.

We present the case of an elderly female patient who presented to us with signs, symptoms, laboratory parameters and radiological features of typical community acquired pneumonia (CAP) not responding adequately to optimum antibiotic therapy. Though her symptoms improved mildly and inflammatory markers normalized, she never achieved her baseline status. A lung biopsy was thus performed which revealed AFOP. The disease was of the sub acute variety and responded well to steroids. She relapsed a few weeks later due to poor compliance with the treatment, however after readmission and retreatment she improved rapidly.

To the best of our knowledge this is the twenty fourth case report on AFOP and the first to be reported from Pakistan and South Asia; moreover our search reveals it to be the 8^th^ case associated with a suspected pulmonary infection.

## Case presentation

A 68 years old female of Afghan descent with a past medical history significant for controlled type 2 diabetes mellitus, spinal stenosis due to which she was bed ridden and recent upper respiratory tract infection presented to the emergency department with progressively worsening shortness of breath, high grade continuous fever associated with rigors and chills, and a cough productive of thick whitish sputum for 2 weeks. She had received 2 courses of antibiotics from a general practitioner without relief.

She did not smoke and had no respiratory or cardiac disease. She had a family history of ischemic heart disease and her sister had recently been treated for pulmonary tuberculosis.

On examination, she was an elderly lady in distress. Her pulse rate was 110/minute, blood pressure 110/70 mm hg, temperature 102 degrees Fahrenheit (°F) and respiratory rate 30/minute. Her oxygen saturation was 92% on room air. The percussion note was dull over the right lower lung zone. On auscultation, air entry was decreased in the right lower lung zone with bilateral coarse pan inspiratory crepitations and increased vocal resonance audible up to the middle lung zones.

Arterial Blood gases revealed a pH of 7.44, partial pressure of oxygen (pO2) 88 mm hg, partial pressure of carbon dioxide (pCO2) 33 mm hg, bicarbonate 22 meq/L and oxygen saturation 93%.

**Figure 1:**
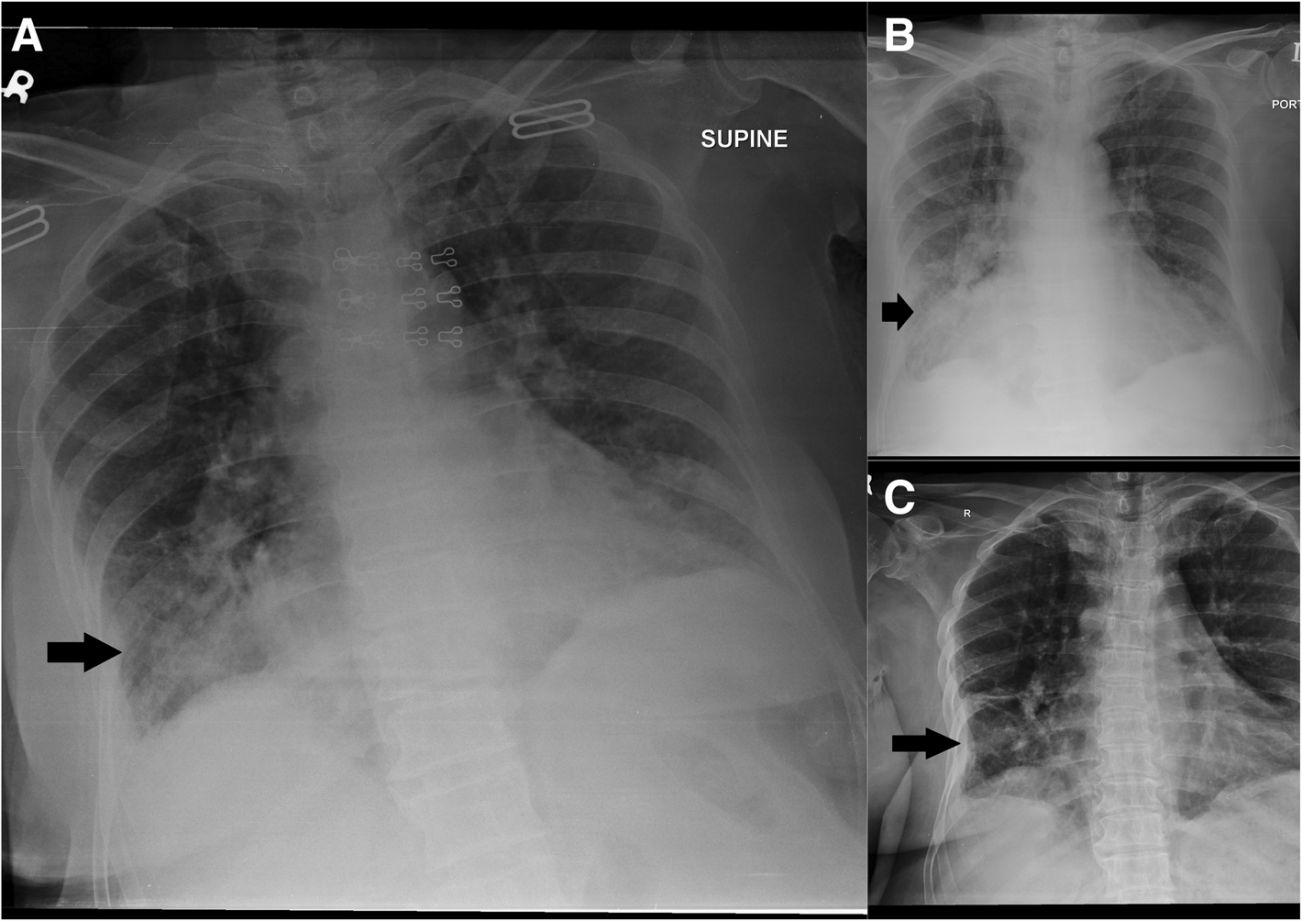
**Chest Radiographs during the first admission. Panel A** shows the chest X-ray on presentation. There is an inhomogeneous opacification with soft tissue infiltrates noted in the right basal lung. There is also soft tissue opacification and haziness in the left lung lower zone. Both costophrenic angles appear hazy. **Panel B** shows the X-ray after a week of antibiotic therapy. There is interval increase in homogenous haze representing air space shadowing in the right lower lung zone. Right costophrenic angle is intervally more blunt. **Panel C** shows the chest x ray after a week of corticosteroid therapy. There is interval resolution of right lower lobe opacity. The rest of the findings are unchanged.

A chest X-ray revealed an inhomogeneous opacification in the right basal lung with slight volume loss accompanied by alveolar and interstitial infiltrates (Figure 1A). The white blood cell count was 30,900 cells/mm^3^ (differentials 90% neutrophils), C reactive protein (CRP) level 294 mg/dL and the Erythrocyte Sedimentation Rate (ESR) was 65 mm/hr. The blood cultures revealed no growth while sputum grew the normal respiratory tract flora. Three consecutive samples for Acid Fast Bacilli (AFB) were also negative. There was no other identifiable source of infection in the body.

Autoimmune work up including anti nuetrophil antibody (ANA), anti nuetrophil cytoplasmic antibody (ANCA), and rheumatoid factor (RF) were all negative.

The patient scored 2/5 on the CURB65 scoring system for community acquired pneumonia and needed hospital admission. She was initially treated with moxifloxacin 400 mg intravenously once daily and intravenous fluids. Serial White cell counts and CRP levels showed resolution with antibiotic therapy; however the patient’s complaints improved minimally. Serial Chest X rays showed no significant resolution of the right lower lung zone opacity (Figure 1B).

**Figure 2:**
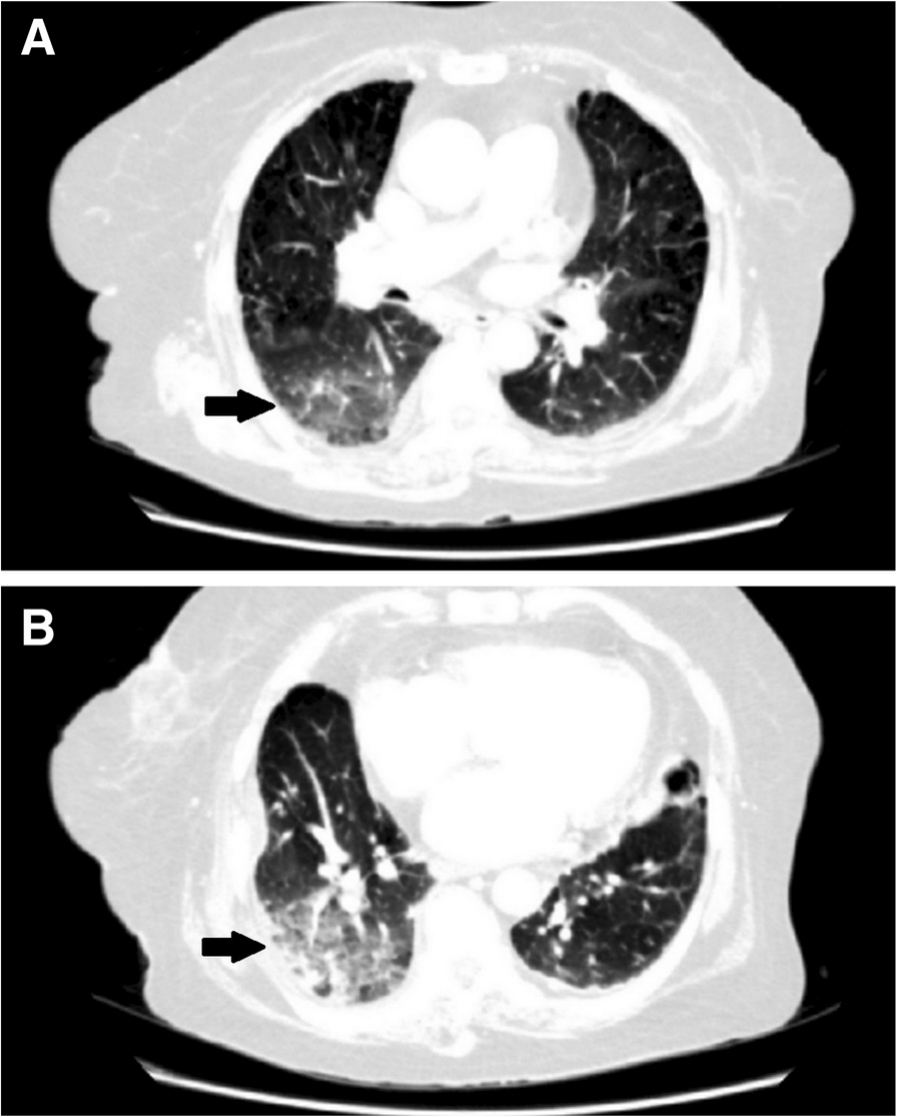
**High Resolution computed tomography scan of the chest. Panel A** and **B** show soft tissue nodular infiltrates and dense consolidation in the right lower lobe of the lung. There is ground glass opacification and haze bilaterally with more prominenence in the right lower lobe. Atelactatic changes are also present right middle lobe, ligular segment and left lower lobe. There is pleural thickening bilaterally and calcified pleural plaques are noted along the diaphragmatic pleura on the left.

A High resolution computed tomography scan (HRCT) was performed 4 days after antibiotic therapy. It revealed soft tissue nodular infiltrates and dense consolidation in the lower lobe of the right lung. There was ground glass opacification and haze bilaterally with more prominenence in the right lower lobe (Figure 2). Atelactatic changes were also present right middle lobe, ligular segment and left lower lobe. There was pleural thickening bilaterally and calcified pleural plaques were noted along the diaphragmatic pleura on the left. Our experienced radiologist described these changes were most probably due to chronic infective/granulomatous disease, most probably tuberculosis (TB). In high suspicion of tuberculosis, especially since the patient was not recovering, had a family history of treated TB in the past one year and since TB is extremely common in our country, anti tuberculous therapy (ATT) was begun empirically.

**Figure 3:**
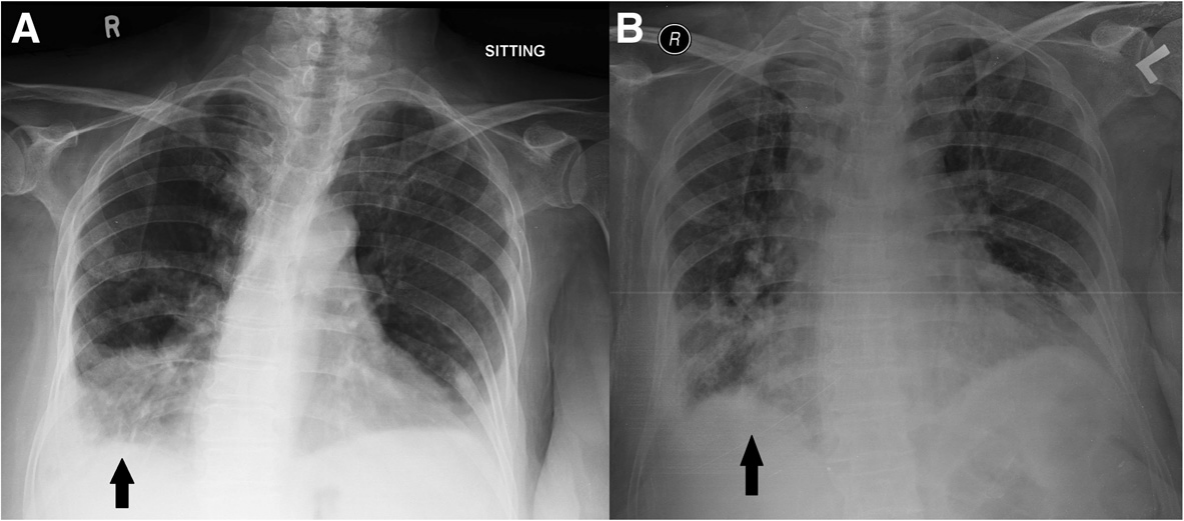
**Chest radiographs during the second admission. Panel A** shows the radiograph after non compliance to corticosteroid therapy*.* There is development of right lung haziness and bilateral pleural effusions with a right lower zone consolidation patch. **Panel B** shows the radiograph on discharge after re treatment with corticosteroids. There is interval resolution of patchy lower zone lung infiltration which was more marked on the right. Improved aeration of the lung is noted.

Despite ATT, patient did not improve. A bronchospcopy was refused by the patient’s family. Thus finally a CT guided biopsy of the right lower lung lobe was planned on the 8^th^ hospital admission day. Histopathology revealed multiple organizing “fibrin balls” within alveolar spaces without associated hyaline membrane formation. Variable areas of spindle cells fibroblastic proliferation were also seen with focal type II pneumocyte hyperplasia. Special stains for fungus, Acid Fast Bacilli (AFB) and gram staining were all negative. There was no growth on culture of the biopsied tissue. These changes are well depicted in Figure 3.

Thus, finally with a diagnosis of acute fibrinous and organizing pneumonia we started methyl prednisolone 60 mg/kg intravenously every six hours. There was a dramatic and excellent response. Patient was discharged after 10 days on oral prednisolone 40 mg daily; at this point she was clinically much better, and very high doses of steroids were avoided due to already severe osteoporosis. Figure 1C shows the interval improvement of the chest x ray findings with this treatment.

One month later she presented again with shortness of breath and non productive cough. She admitted to have stopped taking steroids for the past 10 days. After readmission she was discharged on the same dose of prednisolone with strict advice of adherence to steroids. Her chest radiographs on this admission and discharge are shown in Figure 3. On the next follow up 1 month later, she was completely symptom free. Currently she is on a tapering dose of steroids and is being evaluated by a neurosurgeon for correction of her spinal stenosis. Over all she tolerated the treatment well and complied well after her first exacerbation.

## Discussion

AFOP was first reported in detail in a retrospective analysis of 17 case reports in 2002 [1]. Our search on PubMed revealed 23 cases reported since then of which 3 described AFOP in children and neonates. Qui YY et al. have published a review of 5 cases that were analyzed retrospectively as well [3].

AFOP has been reported in all age groups with an average age of 62 years originally described by Beasely et al. [1]. Later, it was also reported in infants, children and adults [4-6]. Males are affected more commonly [1]. Our patient was an elderly female.

AFOP can present with a variety of signs and symptoms. The most common symptom is shortness of breath which may be accompanied by cough, fever, hemoptysis and other constitutional symptoms [1,6]. Two variants of the disease have been described. A sub acute variety, which does not progress to respiratory failure and has a very good prognosis [1,6]. Our patient suffered from this variant which had an excellent response to corticosteroids. The other variant usually manifests as a fulminant respiratory failure requiring mechanical ventilation. Mechanical ventilation is associated with worse prognosis in AFOP [1,6-8]. On average, severe disease usually causes death within 29 days from symptom onset [1].

AFOP has been described with numerous conditions in the past while twelve reported cases were idiopathic. Some known associations include ankylosing spondylitis, dermatomyositis, polymyositis, undifferentiated connective tissue diseases, SLE with antiphospolipid syndrome, most recently anti synthetase syndrome and primary biliary cirhosis [1,5,9-12]. Thus, there might be a strong association between autoimmune disease and AFOP [13]. There has been a case reported in a patient following bone marrow transplant for acute myelogenous leukemia [6]. Drugs implicated in AFOP include abacavir, amoidarone and decitabine [6,14].

AFOP has also been reported with infective etiologies including Hemophilus Influenza, Acinetobacter Baumannii and Histoplasma species [1]. Otto et al. reported H1N1 causing AFOP in a patient with bilateral lung transplantation. Human immune deficiency virus (HIV) alone and in association with Pneumocystis Jirovecci pneumonia has also been reported [8,14]. Chlamydia pneumonia serology has been found positive in a patient with AFOP [15,16].

We would specially like to discuses AFOP’s association with infection and describe the pattern of disease we encountered in this case. Our patient presented a picture of community acquired pneumonia (CAP). Her CRP and white cell counts were elevated with striking neutrophilia. These lab parameters normalized with antibiotic therapy, however she still remained symptomatic. This prompted us to consider bronchoscopy which the patient’s family refused. To obtain a tissue diagnosis, a CT guided biopsy of the lung lesion as visualized on HRCT was performed which clinched the diagnosis. Such a pattern of disease describing AFOP unmasked by treating a suspected CAP has rarely been described. Thus, our report emphasizes that AFOP should be considered in the differentials of CAP unresponsive to standard antibiotic therapy.

Our case has striking similarities to a few previous cases which heralded such a diagnostic dilemma. These patients with a suspected diagnosis of CAP did not respond to antibiotic therapy, only to reveal AFOP on biopsy and histopathology [15-17].

AFOP can produce an elevated ESR and CRP; however a raised white cell count with striking neutrophilia is most suggestive of a simultaneous bacterial infection which also responded to antibiotic therapy. Though the cultures did not reveal any organism, prospective studies show that the cause of CAP is not identifiable in 40-60% cases, thus cultures may be negative [18]. Previous courses of antibiotics may also have contributed to negative culture results.

We would also like to discuss in the light of past literature, the role of important diagnostic modalities we used in obtaining diagnosis. HRCT provided valuable input regarding the site of the lesions. Many different radiological appearances of AFOP have been described in some review articles. Beasely et al. and Lopez et al. described bilateral “diffuse lung infiltrates” and “bilateral patchy lung infiltrates” respectively, as the most common radiographic abnormalities [1,6]. Our patient had generalized nodular and interstitial infiltrates with dense consolidation and ground glass opacification in the right lower lobe. Though these findings have been described previously, they are less common in AFOP, particularly the pattern of lung lobe or complete lung consolidation [6,17].

Though we could not perform Bronchoscopy in this case, our literature search showed that broncho alveolar lavage (BAL) provided non specific and inconclusive findings in AFOP in almost 100% cases. The only case where it provided a breakthrough in diagnosis was when a trans bronchial biopsy yielded a histological diagnosis [6].

**Figure 4:**
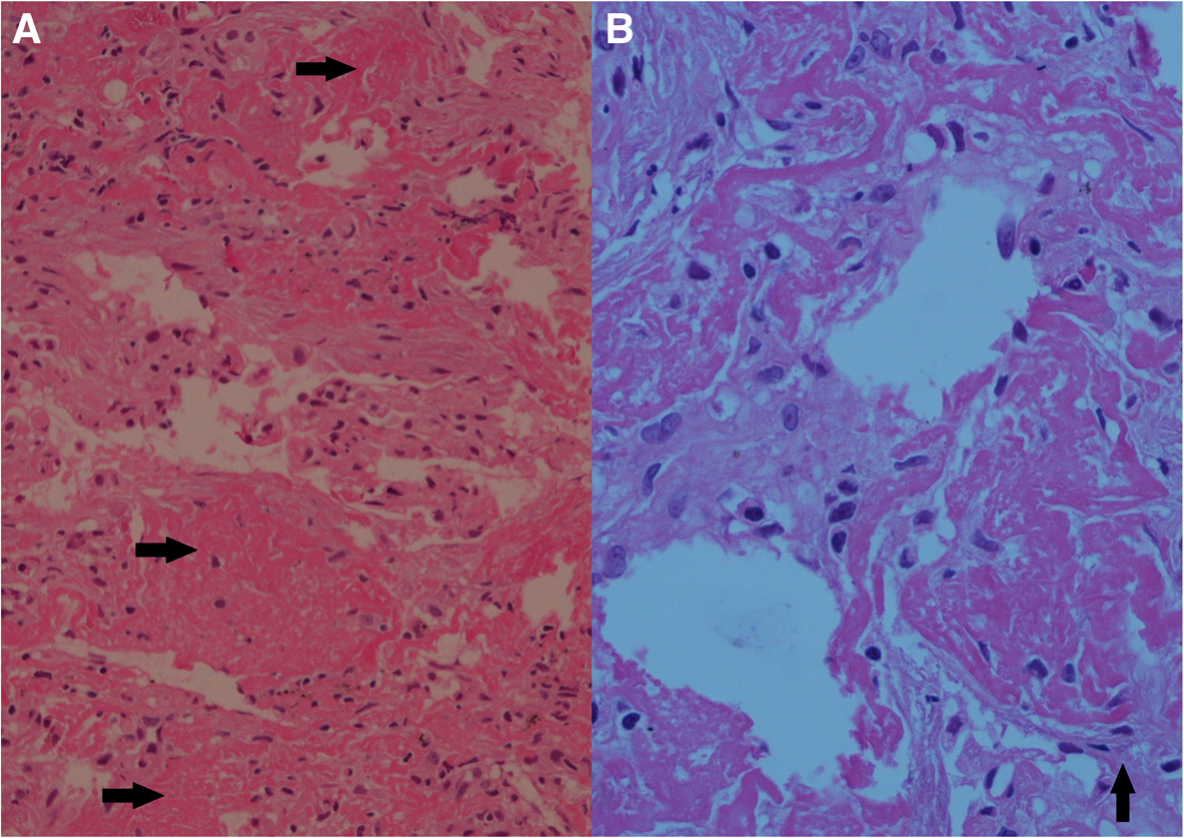
**Histopathology of the lung biopsy. Panel A** shows multiple organizing “fibrin balls” within alveolar spaces without associated hyaline membrane formation. Variable areas of spindle cells fibroblastic proliferation were also seen with focal type II pnuemocyte hyperplasia, the latter being depicted by the black arrows in **panel B**.

Since AFOP is a histological diagnosis, a good tissue sample is essential for examination. The most commonly used methods to obtain a sample include video assisted throacoscopic biopsy (VATS) in living patients and CT guided biopsy. Trans bronchial biopsy has also been used [6]. Histology is classic; the presence of intra alveolar fibrin balls is the hallmark as shown in Figure 4. Organizing pneumonia heralded by presence of proliferating fibroblastic foci (Masson bodies) and type II pneumocyte hyperplasia is also observed [1]. Unlike Diffuse alveolar damage, hyaline membranes are absent [1]. These features are well depicted by our photomicrographs.

Numerous treatment modalities have been used for AFOP. The most common and successful has been corticosteroids. Other agents successfully used include cyclophosphamide, mycophenolate mofetil, azathioprine and mechanical ventilation [6,16]. Different corticosteroid dosages have been used. Our experience was to use intravenous methyl prednisolone 60 mg every 6–8 hourly for the first 5 days followed by gradual tapering and maintenance to a dose of 40 mg daily for 3 months. Unfortunately our patient did not comply with the treatment and relapsed, got readmitted and was managed on the same regimen. Thus, we stress on the importance of adherence to corticosteroid therapy for at least 3 months. Relapse may also occur during tapering of steroids [2,19].

## Conclusion

From this case we conclude, that AFOP is a rare clinical pattern of lung injury and should be considered as a differential in patients with suspected CAP unresponsive to standard antibiotic therapy. Diagnosis is by biopsy and histopathological examination of the obtained tissue. Treatment is with corticosteroids; the response is dramatic and we stress on good compliance with treatment since relapse can occur during tapering steroids or with non compliance.

Numerous conditions have been described with AFOP. Our case is distinct since we describe a pulmonary infection in association with AFOP with slightly atypical radiological features and clinical course.

Finally, AFOP might represent an under diagnosed and under reported condition, especially in the developing countries. This may be due to the complicated means of obtaining a tissue diagnosis which are not present in most tertiary care settings of our region. Further studies are needed to elucidate various clinical aspects of this interesting pattern of lung injury.

## Consent

Written informed consent was obtained from the patient for publication of this Case Report and any accompanying images. A copy of the written consent is available for review by the Editor-in-Chief of this journal.

## Abbreviations

AFOP: Acute fibrinous and organizing pneumonia
CT guided biopsy: Computed tomography guided biopsy
HRCT: High resolution computed tomography
VATS: Video assisted throacoscopic surgery
CAP: Community acquired pneumonia
